# Co-Infection by LF-89-Like and EM-90-Like Genogroups of *Piscirickettsia Salmonis* in Farmed Atlantic Salmon in Chile: Implications for Surveillance and Control of Piscirickettsiosis

**DOI:** 10.3390/pathogens12030450

**Published:** 2023-03-13

**Authors:** Marco Rozas-Serri, Andrea Peña, Ian Gardner, Estefanía Peñaloza, Lucerina Maldonado, Ariel Muñoz, Fernando O. Mardones, Catalina Rodríguez, Ricardo Ildefonso, Carolina Senn, Felipe Aranis

**Affiliations:** 1 Pathovet Labs SpA, Puerto Montt 5480000, Chile; 2 Department of Health Management, Atlantic Veterinary College, University of Prince Edward Island, Charlottetown, PE C1A 4P3, Canada; 3 Escuela de Medicina Veterinaria, Facultad de Agronomía e Ingeniería Forestal, Facultad de Ciencias Biológicas, Facultad de Medicina, Pontificia Universidad Católica de Chile, Santiago 3542000, Chile

**Keywords:** Atlantic salmon, *Piscirickettsia salmonis*, LF-89-like, EM-90-like, co-infection

## Abstract

Piscirickettsiosis (SRS), caused by *Piscirickettsia salmonis*, is the main infectious disease that affects farmed Atlantic salmon in Chile. Currently, the official surveillance and control plan for SRS in Chile is based only on the detection of *P. salmonis*, but neither of its genogroups (LF-89-like and EM-90-like) are included. Surveillance at the genogroup level is essential not only for defining and evaluating the vaccination strategy against SRS, but it is also of utmost importance for early diagnosis, clinical prognosis in the field, treatment, and control of the disease. The objectives of this study were to characterize the spatio-temporal distribution of *P. salmonis* genogroups using genogroup-specific real-time probe-based polymerase chain reaction (qPCR) to discriminate between LF-89-like and EM-90-like within and between seawater farms, individual fish, and tissues/organs during early infection in Atlantic salmon under field conditions. The spatio-temporal distribution of LF-89-like and EM-90-like was shown to be highly variable within and between seawater farms. *P. salmonis* infection was also proven to be caused by both genogroups at farm, fish, and tissue levels. Our study demonstrated for the first time a complex co-infection by *P. salmonis* LF-89-like and EM-90-like in Atlantic salmon. Liver nodules (moderate and severe) were strongly associated with EM-90-like infection, but this phenotype was not detected by infection with LF-89-like or co-infection of both genogroups. The detection rate of *P. salmonis* LF-89-like increased significantly between 2017 and 2021 and was the most prevalent genogroup in Chilean salmon aquaculture during this period. Lastly, a novel strategy to identify *P. salmonis* genogroups based on novel genogroup-specific qPCR for LF-89-like and EM-90-like genogroups is suggested.

## 1. Introduction

Piscirickettsiosis (SRS) is caused by *Piscirickettsia salmonis*, a Gram-negative facultative intracellular bacterium that belongs to the subdivision of gamma-Proteobacteria [1]. *P. salmonis* infection has been detected in the main salmon-producing countries worldwide but is notably more prevalent in farmed salmon in Chile [2]. The most important factors of SRS prevalence are the number of infected farms in upstream waters, followed by seawater salinity and temperature [3]. SRS was responsible for 10.2% of Atlantic salmon (*Salmo salar* L.) and 8.4% of rainbow trout (*Oncorhynchus mykiss*) total mortality production during the first half of 2022 [4].

*P. salmonis* has been identified and described in different parts of the world since the early 1990s [1], but while LF-89-like isolates are closely related to each other, the Chilean EM-90-like isolates are genetically divergent [5,6]. The accumulated evidence regarding genomic structures and phylogenetic relationships currently supports the existence of both *P. salmonis* LF-89 and EM-90 genogroups [7,8,9,10,11,12]. Furthermore, it has been demonstrated that both *P. salmonis* genogroups are widely spread throughout different geographic areas in Chilean salmon aquaculture [9,13,14,15]. In fact, reports demonstrate how the ecological balance of both genogroups changed over time from LF-89-like prevalence between 2010 and 2014, to EM-90-like dominance in 2015 [15] and in 2019 [13]. However, there is no factual evidence to date about the spatio-temporal dynamics of early infection of these genogroups under field conditions, nor any confirmation on the likelihood of co-infection by both genogroups simultaneously in the same fish and/or tissues. A tendency change of the positivity rate caused by both genogroups between 2015 and 2019 could potentially be the underlying reason for pharmaceutical laboratories to decide using only the EM-90-like genogroup in their commercial vaccines based on the assumption that these vaccines would generate cross-immunity also for both genogroups.

However, further evidence indicates that the pathogenesis of SRS in post-smolt Atlantic salmon infected with LF-89-like and EM-90-like isolates can be different [16] when using an experimental model based on infection by cohabitation. Briefly, fish infected with the EM-90-like genotype showed higher and faster cumulative mortality compared to those infected with the LF-89-like genotype. Additionally, while the EM-90-like isolate induced a systemic and hemorrhagic disease characterized by injuries affecting several internal organs and skin ulcers, fish infected with LF-89 showed more classic SRS lesions in liver and kidneys. Furthermore, both LF-89-like and EM-90-like isolates modulated a similar immunological response based on a substantial activation of the innate immune response and a significant inhibition of the T cell-mediated adaptive response. This reaction was especially predominant in EM-90-like infected fish and could potentially explain the higher bacterial loads and more severe tissue lesions, resulting in a lower survival rate [17,18,19]. It has been widely documented that commercially available vaccines for SRS control have demonstrated insufficient efficacy in field conditions [2,17,20,21,22,23,24] and the only commercial live vaccine for SRS control, the current standard for vaccination strategy in the Chilean salmon industry, is based solely on a *P. salmonis* EM-90-like isolate.

Despite all the aforementioned information, the official surveillance and control plan for SRS, which was implemented in 2012 [25], is based only on the disease caused by *P. salmoni*; it does not incorporate detection for either of the genogroups of the etiologic agent of this disease. The general aim of the official surveillance program is to minimize the SRS impact in the country through early detection, following up with infected farms and executing control measures at different stages of the disease in salmon farms. Due to widespread infection among salmon farms located in Los Lagos and Aysén, and the very high likelihood of SRS outbreaks during the production cycle for all farmed salmon species (>90%), epidemiological surveillance of *P. salmonis* at the genogroup level is essential, not only for defining and evaluating vaccination strategies against SRS but also for early diagnosis, clinical prognosis in the field, and treatment and control of the disease.

It is possible that biological mechanisms of both *P. salmonis* genogroups could modulate the interaction with its host, thereby supporting our hypothesis that *P. salmonis* promotes a very complex co-infection that appears to be highly dynamic over time, which poses a major challenge for optimal treatment and control. Additionally, the vaccination strategy based on administrating vaccines formulated only with the EM-90-like genogroup could have caused an increase in the incidence and positivity rate of LF-89-like isolates in recent years. The objectives of this study were as follows: (a) prospective characterization of the spatio-temporal distribution and dynamics of *P. salmonis* genogroup presence within and between seawater farms, as well as individual fish and tissues during early infection in Atlantic salmon under field conditions; (b) retrospective estimation and characterization of the changes in abundance and the positivity rate of both *P. salmonis* genogroups considering laboratory case studies between 2017 and 2021; and (c) knowledge generation to improve the surveillance and control strategy for SRS in Chile. 

## 2. Material and Methods

### 2.1. Prospective Field Survey

Due to logistical and financial reasons, the study was limited to six Atlantic salmon farms located in Los Lagos Region, which were participating in an epidemiological SRS. These marine sites were stocked between May and September in 2018 (Table 1). Fish from all selected farms did not receive antibiotic treatments during the monitoring period. Table 1 shows the entry date to the sea (stock date), the start, the end monitoring date, as well as its length, the cages sampled, and the total number of fish per farm included in the study. On average, five fish were collected from three cages in each farm during the monitoring time (i.e., average of 15 fish per sampling time). 

Prospective salmon sampling was performed by targeting moribund or recently dead fish, and sample size was increased by random sampling of live fish until the minimum sample size was obtained. Fish were individually inspected by complete anatomopathological examination, and the severity of findings was recorded using a standardized semi-quantitative system (Table S1). Tissue samples of approximately 5 mm^3^ from brain, liver, spleen, head-kidney, and hind-kidney were collected from each fish and placed in 2 mL microfuge tubes with 70% *v*/*v* ethanol. Samples were then transported in polyethylene boxes with gelpacks at 2–4 °C and stored at −80 °C in the laboratory until further analysis. Negative samples for *P. salmonis* by qPCR, obtained from farm No. 3 (company B) were kept in the experimental design as negative controls.

### 2.2. Retrospective Field Survey

Three hundred and sixty-three (363) sets of organ samples (liver, kidney, spleen, and brain) from Atlantic salmon farmed in Los Lagos and the Aysén Region in Chile were received in our laboratory for *P. salmonis* detection and genogroup identification by qPCR between 2017 and 2021. All tissue samples were delivered to the laboratory fixed in 70% *v*/*v* ethanol.

### 2.3. RNA Extraction, Quantification, and P. salmonis Detection by qPCR

*P. salmonis* was detected using Taqman^®^-based (Applied Biosystems, Life Technologies, Waltham, MA, USA) qPCR, as previously described by Karatas et al. [26], using primers that bind to the 16S rRNA gene (F: AGGGAGACTGCCGGTGATA; R: ACTACGAGGCGCTTTCTCA; Probe: TGGGGCGTACAGACGGAGGC; Efficiency: 2.05; R^2^: 0.9999). These primers have previously been used in both field [27,28] and experimental (16–19) conditions. Hereafter, we will refer to this assay as “universal” *P. salmonis* qPCR. This assay detects both LF-89-like and EM-90-like isolates in the Chilean salmon industry, as the 16S rRNA gene is conserved between both genogroups. Genomic DNA was extracted from brain, liver, spleen, head-kidney, and hind-kidney of each fish using the EZNA Tissue DNA kit (Omega Bio-Tek Inc., Norcross, GA, USA) according to the manufacturer’s instructions. The qPCR was performed using a total volume of 15 μL for each sample, containing 2 × KAPA PROBE FAST qPCR Master Mix Universal, 300 nM of each primer, 200 nM of probe, 0.3 μL of 50 × ROX High, and 2 μL DNA of each sample. The qPCRs were carried out in the StepOne Plus Real-Time qPCR System (Applied Biosystems, Life Technologies, Waltham, MA, USA) using the following parameters: 95 °C for 3 min for initial denaturation, 95 °C for 1 s, and 60 °C for 20 s for 40 cycles. A positive control (*P. salmonis* DNA), a negative control without DNA, and a negative extraction control were also included in every run. Cycle threshold (Ct) values were recorded (run to a maximum of 40 Ct), and a Ct of <33.01 was considered positive, and negative if otherwise, based on what we have described previously [28].

### 2.4. Genogroup-Specific PCR to Discriminate the LF-89-Like and EM-90-Like

To discriminate between LF-89-like and EM-90-like genogroups, we designed a TaqMan^®^-based (Applied Biosystems, Life Technologies, Waltham, MA, USA) qPCR to amplify the most genetically diverse region of the 16S rRNA gene. Conserved primers to amplify both LF-89-like and EM-90-like (Forward: CGATAAGTTGACCGCCTGGG; and Reverse: CTCTAAGCTCTCCCGAAGGCAC) were used, but different probes were applied to discriminate between genogroups (LF89-Probe: TTCCGCATCTCTCTCTGCAGAA; EM90-Probe-ATCAATATATCTCTCTCTCTATCAAC). Amplification efficiencies for each qPCRs were calculated using serial dilutions of a positive sample and the equation E = 10^(1/slope), resulting in 1.95 and R^2^ 0.9998 for LF-89-like and 1.98 and R^2^ 0.9995 for EM-90-like. We worked with sixty-seven (67) fish having positive “universal” *P. salmonis* qPCR results. Briefly, the same DNA extracted in the previous step was used to perform genogroup-specific qPCR. The PCRs were performed using a total volume of 15 µL for each sample, containing 2 × KAPA PROBE FAST qPCR Master Mix Universal, 200 nM to 400 nM primers (depending on the PCR set), 200 nM to 400 nM probe (depending on the PCR set), 0.3 µL of 50 × ROX High, and 2 µL DNA of each sample. The PCRs were carried out in the StepOne Plus Real-Time PCR System (Applied Biosystems, Life Technologies, Waltham, MA, USA) using the following program: 95 °C for 3 min for initial denaturation, 95 °C for 1 s, and 60 °C for 20 s for 40 cycles. A positive control (*P. salmonis* LF-89-like and EM-90-like DNA), a negative control without DNA, and negative extraction controls were also included in every run.

Genogroup-specific qPCR were validated according to the Manual of Diagnostic Tests for Aquatic Animals of the World Organization for Animal Health (WOAH, France, Paris; https://www.woah.org/en/what-we-do/standards/codes-and-manuals/aquatic-manual-online-access/; accessed on 15 January 2023). For the implementation of qPCRs, isolates of each genogroup of *P. salmonis* previously identified as LF-89-like and EM-90-like were acquired from the national reference laboratory for SRS in Chile. The specificity of the LF-89-like and EM-90-like genogroup-specific qPCRs was evaluated using DNA extracted from their respective colonies grown on Austral-TSHem agar [29]. Subsequently, specificity tests were performed that demonstrated the absence of cross-reactions with enzootic pathogens in Chile such as *Renibacterium salmoninarum*, *Aeromonas salmonicida* atypica, *Flavobacterium psychrophylum* and *F. columnare*, *Tenacibaculum maritimum* and *T. dicentrarchi*, *Neoparamoeba perurans*, Infectious Salmon Anemia virus (ISAV), Infectious Pancreatic Necrosis virus (IPNV), and Piscine Ortho-reovirus (PRV). The Ct value were recorded (run to a maximum of 40 Ct), and a Ct under the cut-off point was considered positive, and negative if otherwise. Cut-off points for the genogroup-specific assays were 34.54 (LF-89-like) and 33.37 (EM-90-like).

The lowest Ct value for EM-90-like and LF-89-like was also calculated across all 5 tissues sampled (brain, spleen, liver, head-kidney, and hind-kidney) for fish classification as either infected with both EM-90-like and LF-89-like (Ct values < 33.37 for EM-90-like and Ct value < 34.54 for LF-89-like), infected with EM-90-like only (Ct value < 33.37 for EM-90-like and Ct value ≥ 34.54 for LF-89-like), infected with LF-89-like only (Ct value < 34.54 for LF-89-like and Ct value ≥ 33.37 for EM-90-like), and infected with neither genogroup (Ct values ≥ 33.37 for EM-90-like and Ct value ≥ 34.54 for LF-89-like). All fish/tissues tested positive for *P. salmonis* were also negative for *Renibacterium salmoninarum* using a qPCR assay described by Suzuki and Sakai [30], a bacterium that could induce gross pathology like that of *P. salmonis*.

### 2.5. Data Management and Statistical Analysis

The frequency of genogroup qPCR positive results at the fish level was cross tabulated by farm (both, EM-90-like, EM-89-like, or neither) and at the tissue level for EM-90-like and EM-89-like positive test results (range, 0 to 5 positive tissues). For farms with mixed infections, McNemar’s test for correlated proportions was used to test whether the percentages of qPCR positive results differed significantly (*p* < 0.05) between genogroups. Odds ratios and 95% confidence intervals were calculated to measure the strength of association between *P. salmonis* genogroup and gross pathology. These analyses were restricted to visible pathological findings with at least 10 cases in moderate or severe categories, as few cases would have resulted in low statistical power. Data management and analyses were done in MedCalc^®^ Statistical Software version 20.215 (MedCalc Software Ltd., Ostend, Belgium); https://www.medcalc.org; accessed on 28 January 2023).

## 3. Results

### 3.1. The Spatio-Temporal Distribution of LF-89-Like and EM-90-Like Is highly Variable within and between Seawater Farms

The percentage of LF-89-like and EM-90-like positivity was highly variable among the six sites studied and within each site. Fish from farms 1 and 2 (company A) (Table 2) showed the first positive qPCR result 111 days (average weight 775 g) and 197 days (2486 g) after being stocked in salt water, respectively. The time elapsed between the stock date and the first detection in farms 5 and 6 (company C) (Table 3) was 92 days (690 g) and 141 days (1026 g), respectively. Finally, the farms of company B (Table 4) showed positivity to *P. salmonis* at the earliest and latest stages of the study; while farm 4 presented the earliest first positivity of the farms studied (83 days after stock and 611 g), farm 3 did not show positive fish until the end of the study period (176 days and 1948 g) and remained as a control farm without SRS outbreaks. In addition, 74.6% (50/67) of the positive *P. salmonis* genogroup-specific PCR results were from moribund/dead fish, while 25.4% (17/67) were from live fish.

### 3.2. P. salmonis Infection Is Multi-Genogroup (Co-Infection) at Farm, Fish, and Tissue Levels

Three (# 2, 4, and 6) of the five *P. salmonis* farms in the prospective study revealed evidence of co-infections with EM-90-like and LF-89-like genogroups at farm level, and two of the farms with mixed infections also had fish infected with both genogroups (seven in farm 2 and two in farm 6). Farm 1 only showed one positive sample to the EM-90-like genogroup, while farm 5 revealed only positives to the LF-89-like genogroup (n = 19) (Table 5). In farm 6, infection was detected only with EM-90-like (n = 1) and only by LF-89-like (n = 3), but no co-infection with both genogroups of *P. salmonis* was detected (Table 5). There were two fish with CT values > 33.37 for both genogroups and were considered negative (“Neither”). In farm 2, infection with EM-90-like (n = 11) and with LF-89-like (n = 4) was detected, but co-infection with both genogroups was also recorded (n = 7) (Table 5). As a result, the EM-90-like genogroup was detected in 81.8% (18 of 22) of the positive fish at the farm, while the LF-89-like genogroup was detected in 50% (11 of 22) (McNemar *p* = 0.119). In farm 4, infection was only with EM-90-like (n = 19) and co-infection of this with LF-89-like (n = 2), but no infection with LF-89-like alone was recorded (Table 5). In summary, the EM-90-like genogroup was detected in 89.5% (17 of 19) of the positive fish compared with 15.8% (3 of 19) for the LF-89-like genogroup (McNemar *p* = 0.0005).

**Figure 1:**
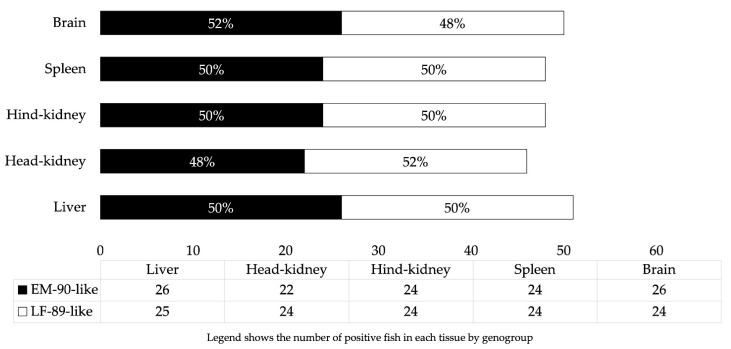
The frequency of qPCR positive results for EM-90-like and LF-89-like (n = 67 fish) at the organ level.

The largest proportion of fish infected with EM-90-like (65.2%) and/or LF-89-like (62.6%) showed no gross pathology (score 0) or showed mild lesions (score ≤ 2) (Table 6). However, 34.3 and 37.4% of EM-90-like and/or LF-89-like infected fish showed moderate to severe pathological lesions (score > 2), respectively (Table 6). Therefore, there was a high probability of early detection of the pathogen and even discriminated its genogroup from asymptomatic fish or fish with mild gross pathology. Finally, no difference was detected in terms of frequency of EM-90-like and LF-89-like genogroups in liver, head-kidney, hind-kidney, spleen, and brain of the 67 qPCR+ fish (Figure 1). However, while the frequency of EM-90-like was slightly higher in brain, LF-89-like detection was marginally higher in head-kidney (Figure 1). The ranges of qPCR positive tissues were 22 to 26 and 24 to 26 for EM-90-like and LF-89-like, respectively.

### 3.3. Liver Nodules Are Associated with EM-90-Like Infection, but Not with LF-89-Like or with Co-infection of Both Genogroups

Most fish infected with EM-90-like (67.2%) and/or LF-89-like (70.2%) showed no macroscopic lesions at necropsy (score = 0) or had mild lesions (score = 1) because fish were in the early stage of infection (Table S2). Of the 12 gross pathology considered for genogroup association analysis, only five (liver nodules, splenomegaly, renomegaly, hepatomegaly, and brain abnormalities) were found to have at least 10 cases of moderate (score = 2) or severe lesions (score = 3) and for brain samples, hyperemia (score = 2) or hemorrhage. Liver nodule was the only lesion among the five that had a *p* value of <0.05, indicating a causative association with statistical significance (Table 7). Excluding the nine fish with both genogroups, 12 of 13 fish with liver nodule scores of 2 or 3 had positive qPCR results for EM-90-like compared with a single fish for EM-89-like positive results. The odds for liver nodules (scores 2 and 3) was 19.5-fold greater (95% confidence interval = 2.3 to 164.6) for EM-90-like infections compared with EM-89-like infections. For the nine fish with both genogroups, the odds for liver nodules was not significantly higher (3.7, 95% confidence interval = 0.2 to 67.2) than the odds for LF-89-like infections.

### 3.4. The Positivity Rate of P. salmonis LF-89-Like Increased Significantly between 2017 and 2021, Being the Main Circulating Genogroup in Chilean Salmon Farming during This Time Period

**Figure 2:**
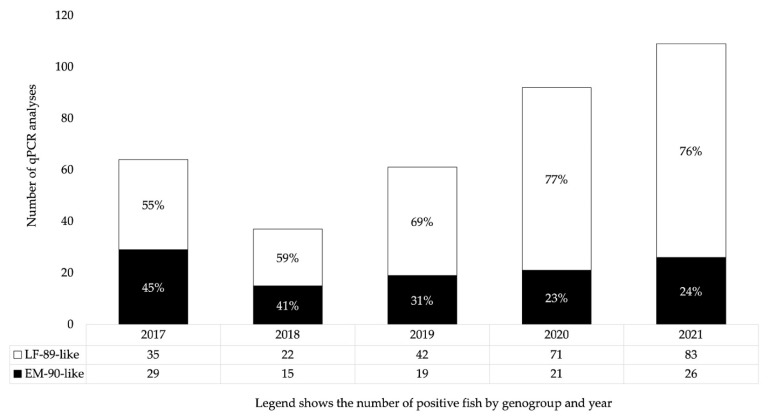
Comparative positivity rate for LF-89-like and EM-90-like genogroups of *P. salmonis* between 2017 and 2021 using genogroup-specific qPCR (n = 363).

The distribution of Atlantic salmon analyzed for *P. salmonis* genogroup identification between 2017 and 2021 is presented in Figure 2. Although the positivity rate of LF-89-like was higher than that of EM-90-like in 2017 (55% LF-89-like vs. 45% EM-90-like), its percentage gradually increased year by year until approximately three out of four “universal” positive *P. salmonis* qPCR results were of the LF-89-like genogroup in 2020 and 2021.

## 4. Discussion

Government authorities and the salmon industry in Chile designed and implemented a specific surveillance and control program for piscirickettsiosis in 2012 [25]. A critical aspect for control is to promote early detection of infection for timely treatment [2,31,32]. Timely diagnosis and training in necropsies are fundamental aspects to reduce the incidence rate and severity of SRS and to optimize control of the disease [33]. The current SRS surveillance program is based on risk-based sampling [34] of five moribund or dead fish from two to three netpens, is cost-effective, and gives a high probability of detection of *P. salmonis* in Atlantic salmon farms in Chile at both the netpen and farm levels [27]. In addition, qPCR is fit for the purpose of presumptive diagnosis and surveillance for the detection of *P. salmonis* cases in endemically infected regions of Chile [28].

Our prospective results showed that the first detection of *P. salmonis* by qPCR was recorded between 83 days (farm 4) and 197 days (farm 2) after the seawater phase started, which coincides with the interval between 30 and 180 days typically described in retrospective epidemiological studies [23,35,36]. *P. salmonis* infection can settle early in the seawater phase, adversely affecting fish health and requiring early antibacterial treatment [37]. However, *P. salmonis* LF-89-like and EM-90-like field isolates have genomic differences that may induce different degrees of virulence [7,11], pathogenesis [16], and immune response [13,17,18,19,38,39,40]. Consequently, epidemiological surveillance of *P. salmonis* should be complemented by the identification of the genogroup mainly involved, although both genogroups are probably concomitant in different organs of the same fish, or that genogroup might prevail at a specific time of infection reflecting a different phase of infection progression. Deeper knowledge is surely required regarding the co-infection of both genogroups, e.g., fish experimentally infected with EM-90-like and L-89-like at the same time and same tanks using the cohabitation model.

The current SRS surveillance and control program considers the 30 days for beginning sampling after the last sea-cage of the farm is completed, meaning that it could usually take up to 3 months to start surveillance. Although the salmon farmers are free to start surveillance voluntarily as early as they deem appropriate, several variables such as the increase in salinity and sea temperature, production scale-up and management, the increase in the incidence and prevalence of gill diseases and the increase in the immunological challenge of farmed fish, the increase in frequency of low-oxygen and microalgae bloom events, and the relative efficacy of the current vaccination strategy based solely on the EM-90-like isolates would support the idea of implementing the monitoring of *P. salmonis* and its genogroups as soon as possible after stocking fish at seawater farms. Indeed, the results of this study demonstrate that even early detection of *P. salmonis* genogroups in multiple organs/tissues is very likely in live, asymptomatic fish.

It is known that *P. salmonis* LF-89-like isolates obtained from different parts of the world are closely related to each other, but the Chilean EM-90-like isolates are unique and genetically divergent from the others [5,6]. In addition, Rozas-Serri et al. [16] demonstrated that the pathogenesis of SRS caused by EM-90-like or LF-89-like genogroup is different, and they have different incubation times under the same experimental conditions (15 and 20 days, respectively). Saavedra et al. [15] showed that all EM-90-like isolates studied were susceptible to quinolones, florfenicol, and oxytetracycline, but most LF-89-like specimens showed resistances to at least one of the antibiotics tested. This correlates well with what we observed in field conditions, since the best therapeutic results for SRS are recorded when the infection is caused by EM-90-like. Taken together, we believe that early detection of the *P. salmonis* genogroups in the seawater farms could work as an indicator of clinical prognosis of the disease and treatment efficacy.

Due to the importance of the species and the fact that it is susceptible to both genogroups of *P. salmonis* [16] we basically studied the field epidemiological situation of both genogroups in Atlantic salmon. While Aravena et al. [13] described that 94.4% of the samples they studied were obtained from moribund fish, our results show that 75% of the positives samples were obtained from moribund fish and 25% from live fish, which is not only significantly lower but would also support that early diagnosis under a weekly monitoring program would provide valuable information from randomly sampled live fish. In addition, fish infected only with EM-90-like showed a higher percentage of liver nodules than those infected only with LF-89-like or co-infected fish, which is consistent with what was reported by Rozas-Serri et al. [16] in Atlantic salmon experimentally infected with both genogroups, as they observed multifocal yellowish-white focal nodules approximately 1–2 mm in diameter in the liver in 6 and 14% of fish infected with LF-89-like and EM-90-like during the early phase of infection, respectively. Our results showed the detection of *P. salmonis* genogroups in different organs/tissues, which confirmed the systemic nature of the infection described previously [2].

Saavedra et al. [15] described a significantly higher *P. salmonis* EM-90-like positivity rate compared to LF-89-like in 2015, indicating a tendency change in comparison to the period 2010–2014, when LF-89-like genogroup isolates were predominant. According to Aravena et al. [13], *P. salmonis* EM-90-like was detected more frequently in the Chilean salmon industry in 2019, although only field samples collected in a short period of time were used, and they did not consider the comparative spatio-temporal aspect or the dynamics of infection in the field. On the contrary, these results do not agree with ours because in 2019 the ratio was 69 and 31% for LF-89-like and EM-90-like, respectively. We speculate that the main reason to explain these differences is that we considered a different number of isolates from geographic areas with different incidence rates and infection prevalences of both genogroups, and we considered only Atlantic salmon as the host.

Our results demonstrate not only that Atlantic salmon can be infected by both genogroups separately at a given time and in each farm, but that there is co-infection with isolates of both genogroups in the same and different tissues/organs of the same fish at the same time. To our knowledge, this is the first description to date of a complex co-infection with both genogroups of *P. salmonis* at the farm, fish, and even tissue levels in farmed Atlantic salmon in Chile. Furthermore, our results demonstrate a change in the tendency of the positivity rate ratio of both genogroups, as LF-89-like diagnoses became significantly more frequent than EM-90-like diagnoses in 2020 and 2021. Then, three out of four “universal” positive qPCR results for *P. salmonis* during 2020–2021 were LF-89-like when using the genogroup-specific PCR assays described here. Therefore, genogroup-specific PCR assays for *P. salmonis* of the type described in this study could be of great practical utility for further epidemiological surveillance of *P. salmonis* at the genogroup level in Chile. Indeed, there are previous reports of alternative laboratory assays that could also be used for the purposes described [14,15].

Precisely in the last two years we have found increased SRS susceptibility of farmed Atlantic salmon, basically in terms of time of first detection and outbreak, increases in the number of therapies, increases in associated mortality, increments in the frequency of therapeutic failures, among other indicators. Although we know that current vaccines do not activate the cell-mediated adaptive immune response necessary to protect and control a facultative intracellular bacterium such as *P. salmonis* during the entire production cycle in the sea [20,21,24,39], we recognize that vaccines play an important role in the current relative control of the disease. The first live attenuated vaccine entered the market in 2016, but it was based solely on an EM-90-like isolate. From 2017 to date, the vaccination strategy based on this vaccine, used along a pentavalent vaccine whose *P. salmonis* component is also EM-90-like but inactivated, has grown to become the current industry standard. Thereby, a biological process might have started in 2017 that could have promoted an increase in the infection pressure of LF-89-like isolates. *P. salmonis* LF-89-like and EM-90-like belong to the same species but are different isolates that could even present different antigens [11,15].

Consequently, it is essential that all commercial vaccines available for SRS control in Chile be formulated with LF-89-like and EM-90-like isolates or their respective antigenic components to induce a “broad spectrum” immune response against *P. salmonis*. Then, knowledge of the spatio-temporal dynamics of *P. salmonis* genogroups in the industry is fundamental for an adequate design of the vaccination strategy for SRS control, and the best way to achieve this is to promote active and passive epidemiological surveillance of *P. salmonis* at the EM-90-like and LF-89-like genogroup levels that works as an early warning indicator regarding possible variation in the incidence and prevalence of both genogroups that supports timely interventions.

## 5. Conclusions

Our study findings demonstrated that the spatio-temporal distribution of LF-89-like and EM-90-like is highly variable within and between seawater farms, and the *P. salmonis* infection is often a complex co-infection that is a multigenogroup at the farm, fish, an even tissue level. The positivity rate of *P. salmonis* LF-89-like increased significantly between 2017 and 2021, being the main circulating genogroup in the Chilean salmon industry. Finally, a strategy to identify *P. salmonis* genogroups based on novel genogroup-specific qPCR for LF-89-like and EM-90-like was reported. Consequently, our results help to better understand the biological interaction of *P. salmonis* and the host and generate knowledge to improve the surveillance and control strategy for SRS in Chile.

## Figures and Tables

**Table 1:** Atlantic salmon seawater farms included in the study, date of entry to the farm and start date of weekly monitoring, total number of weeks under monitoring, cages, and total number of fish sampled.

Company	Site	Date of Entry SW	Start Date Monitoring	End Date Monitoring	Identification of Sampled Cages	Total Number of Weeks	Total Number of Fish
A	1	30.08.2018	25.09.2018	04.01.2019	2-10-11-15	14	210
	2	30.05.2018	06.07.2018	31.01.2019	2-6-7-14	23	330
B	3	28.08.2018	29.09.2018	20.02.2019	4-6-13	26	378
	4	21.09.2018	17.10.2018	01.01.2019	1-2-3-7-8-9-17-18	10	148
C	5	26.09.2018	18.10.2018	06.02.2019	1-3-4-6-7-9	25	164
	6	02.08.2018	17.08.2018	01.03.2019	1-2-3-4-8	11	372

**Table 2:** Results of universal *P. salmonis* qPCR and genogroup-specific EM-90-like and LF-89-like qPCRs at the fish and organ level at site 1 and 2 (Company A).

Company	Site	Date of Entry SW	Follow-Up Start Date	Date to First PCR+	No. Days to First PCR+	Weight (g) at First PCR+	Cage	Dead/Live Fish	qPCR *P. salmonis* (Ct)					qPCR EM-90 (Ct)					qPCR LF-89 (Ct)				
									Spleen	Brain	Liver	Head-Kidney	Hind-Kidney	Spleen	Brain	Liver	Head-Kidney	Hind-kidney	Spleen	Brain	Liver	Head-Kidney	Hind-Kidney
A	1	30.08.2018	25.09.2018	19.12.2018	111	775	110	D	26.72	28.80	22.88	28.66	30.03	28.67	31.49	24.17	30.12	33.00	-	-	-	-	-
	2	30.05.2018	06.07.2018	13.12.2018	197	2486	107	D	24.79	34.22	23.58	26.20	23.30	-	-	-	-	-	25.54	34.10	22.92	25.35	23.74
							107	D	36.52	37.41	36.38	35.57	31.98	-	-	-	-	-	35.67	-	39.38	35.70	31.36
							114	L	-	37.18	25.31	-	-	-	-	25.62	37.09	-	-	-	-	-	-
							114	D	19.76	30.56	36.32	22.83	24.32	20.26	30.87	-	23.39	24.20	-	-	-	31.26	-
				21.12.2018			102	D	36.73	29.42	34.70	35.98	33.50	-	29.89	39.75	33.55	33.83	-	-	-	-	-
							102	D	35.34	31.83	-	32.74	36.12	-	31.92	-	32.91	-	-	-	-	-	-
							107	D	23.14	25.79	18.56	22.77	22.75	23.01	26.10	18.73	23.16	23.03	-	-	-	-	-
							107	D	33.62	32.37	33.02	33.68	31.36	33.17	-	32.81	38.97	33.18	-	-	-	-	-
				10.01.2019			102	D	33.20	31.79	34.79	33.23	31.94	36.52	33.26	-	-	-	33.27	32.92	35.23	32.04	31.41
							107	D	25.63	30.04	24.18	25.29	28.51	-	-	-	-	-	25.85	30.38	24.77	25.35	28.61
							107	D	32.83	33.86	32.24	33.34	33.40	-	-	-	-	-	32.61	34.08	32.96	33.03	33.52
				24.01.2019			102	L	-	-	31.92	35.20	25.55	-	-	32.25	35.24	-	-	-	-	-	24.89
							102	D	28.70	25.85	27.71	25.66	35.52	32.59	26.56	28.94	32.05	38.38	28.61	-	28.22	27.15	36.92
							102	D	24.35	24.48	30.05	30.24	29.72	27.39	25.43	29.09	34.99	32.54	24.71	-	29.38	32.57	31.45
							102	D	20.55	25.54	24.39	25.58	23.30	-	28.17	-	-	-	20.39	26.00	24.16	29.00	25.68
							107	D	21.96	33.03	22.63	26.79	24.08	22.71	33.48	22.94	29.93	24.36	-	36.38	-	-	-
							107	D	26.34	28.10	20.83	27.32	29.90	26.76	28.42	21.42	27.94	30.14	-	-	-	-	36.60
				31.01.2019			102	D	23.52	26.25	18.89	23.50	23.90	23.01	26.10	18.73	23.16	23.03	-	-	-	-	-
							102	D	32.96	28.26	32.78	34.45	31.73	33.17	-	32.81	38.97	33.18	-	-	-	-	-
							114	L	-	-	32.91	32.63	-	-	-	32.89	32.25	-	-	-	-	-	-
							114	D	28.17	30.21	20.96	24.95	25.73	28.27	31.44	21.51	25.53	25.58	-	-	-	-	-
							114	D	36.07	36.57	29.68	37.07	-	37.79	35.10	29.95	-	-	-	-	-	-	-

Ct: cycle threshold; (-): no amplification.

**Table 3:** Results of universal *P. salmonis* qPCR and genogroup-specific EM-90-like and LF-89-like qPCRs at the fish and organ level at site 3 and 4 (Company B). Ct: cycle threshold; (-): no-amplification.

Company	Site	Date of Entry SW	Follow-Up Start Date	Date to First PCR+	No. Days to First PCR+	Weight (g) at First PCR+	Cage	Dead/Live Fish	qPCR *P. salmonis* (Ct)					qPCR EM-90 (Ct)					qPCR LF-89 (Ct)				
									Spleen	Brain	Liver	Head-Kidney	Hind-Kidney	Spleen	Brain	Liver	Head-Kidney	Hind-Kidney	Spleen	Brain	Liver	Head-Kidney	Hind-Kidney
B	3	28.08.2018	25.09.2018	-	-	-	404	-	-	-	-	-	-	-	-	-	-	-	-	-	-	-	-
							406	-	-	-	-	-	-	-	-	-	-	-	-	-	-	-	-
							413	-	-	-	-	-	-	-	-	-	-	-	-	-	-	-	-
	4	21.09.2018	17.10.2018	12.12.2018	83	611	109	D	32.37	34.97	33.15	-	35.52	-	-	-	-	-	-	-	-	-	-
							109	D	31.70	36.90	31.52	30.74	33.54	34.47	-	-	-	-	-	-	33.12	-	-
							117	D	24.82	31.19	18.73	23.51	23.22	25.55	31.33	20.24	28.37	23.93	-	-	-	-	-
							117	D	22.43	27.98	20.33	24.77	24.93	23.45	29.42	21.48	26.36	25.89	-	-	-	-	-
							117	D	20.79	24.79	19.03	21.14	23.74	22.58	25.87	20.08	23.38	32.69	-	-	-	-	-
				19.12.2018			117	D	26.10	28.77	22.32	37.83	25.74	29.77	30.14	24.14	-	27.22	-	-	-	-	-
				26.12.2018			101	D	22.14	28.21	21.35	23.08	19.48	22.92	28.29	22.02	23.39	19.73	-	-	-	-	-
							117	L	22.97	-	35.97	-	36.17	23.32	-	-	30.15	-	-	-	37.18	-	-
							117	L	24.29	34.04	32.44	-	28.88	24.75	-	-	-	28.99	-	34.00	31.62	-	-
							117	L	22.76	-	-	-	31.98	23.60	-	-	-	31.28	-	-	35.23	-	-
							117	L	32.21	37.00	37.00	36.40	33.17	32.47	37.65	-	-	32.62	-	-	-	-	-
							117	D	34.66	30.03	25.35	23.30	25.34	33.11	30.19	26.29	23.35	26.35	-	-	-	-	32.89
							117	D	35.15	28.94	19.28	21.57	22.49	35.27	29.37	19.58	22.05	23.09	-	-	-	-	-
							117	D	32.88	28.56	18.83	21.33	26.12	32.78	28.76	19.25	21.96	27.23	-	-	-	-	-
				02.01.2019			109	D	35.17	30.52	37.04	-	35.49	34.89	30.27	-	-	-	-	-	-	-	-
							117	L	-	22.39	27.69	31.36	-	-	22.67	28.26	31.76	-	-	-	-	-	-
							117	D	-	-	18.63	26.06	23.54	-	-	19.44	26.83	23.48	-	-	-	-	-
							117	D	25.62	28.43	28.41	36.59	31.44	25.03	28.91	28.90	-	30.86	-	-	-	-	-
							117	D	32.70	32.52	18.79	23.35	24.94	33.29	32.08	19.83	23.80	24.82	-	-	-	-	-

**Table 4:** Results of universal *P. salmonis* qPCR and genogroup-specific EM-90-like and LF-89-like qPCRs at the fish and organ level at site 5 and 6 (Company C). Ct: cycle threshold; (-): no-amplification.

Company	Site	Date of Entry SW	Follow-Up Start Date	Date to First PCR+	No. Days to First PCR+	Weight (g) at First PCR+	Cage	Dead/Live Fish	qPCR *P. salmonis* (Ct)					qPCR EM-90 (Ct)					qPCR LF-89 (Ct)				
									Spleen	Brain	Liver	Head-Kidney	Hind-Kidney	Spleen	Brain	Liver	Head-Kidney	Hind-Kidney	Spleen	Brain	Liver	Head-Kidney	Hind-Kidney
C	5	26.09.2018	18.10.2018	27.12.2018	92	690	101	D	26.01	22.49	20.60	25.00	25.97	-	-	-	-	-	26.67	22.81	20.80	25.19	26.10
							109	D	31.78	30.82	35.77	-	32.93	-	-	-	-	-	31.92	30.73	39.60	-	32.27
				09.01.2019			101	L	37.15	32.99	32.03	36.63	-	-	-	-	-	-	-	32.42	31.44	36.15	-
							101	L	36.36	34.16	32.12	34.03	37.06	-	-	-	-	-	34.56	33.39	30.87	33.91	36.38
							101	L	37.00	29.68	-	-	-	-	-	-	-	-	-	28.16	35.24	-	35.64
							101	D	24.88	29.52	20.65	24.98	23.47	-	-	-	-	-	23.65	28.61	19.70	23.96	22.07
							101	D	30.32	19.01	27.50	26.30	30.08	-	-	-	-	-	28.85	18.10	26.52	24.91	28.74
							106	D	34.72	34.76	37.45	35.49	36.49	-	-	-	-	-	35.97	33.89	-	34.52	39.48
				16.01.2019			109	D	33.92	29.15	33.95	33.61	33.31	-	-	-	-	-	32.64	29.28	-	34.13	33.49
				24.01.2019			101	D	23.60	29.42	19.79	28.93	27.04	-	-	-	-	-	23.88	-	19.45	30.67	28.36
				06.02.2019			101	L	32.47	31.02	30.20	35.98	33.96	-	-	-	-	-	32.53	30.99	30.18	-	34.41
							101	L	32.00	34.52	31.65	32.57	31.05	-	-	-	-	-	31.95	33.31	32.09	32.88	31.05
							106	D	27.96	23.88	24.10	25.07	26.57	-	-	-	-	-	27.81	24.26	23.91	25.16	26.99
							106	D	25.95	24.87	27.06	29.10	28.72	-	-	-	-	-	25.83	25.23	27.25	29.09	28.28
							106	D	29.85	20.47	27.62	27.69	29.93	-	-	-	-	-	29.90	20.42	28.00	27.93	29.75
							109	L	-	34.22	29.62	32.21	35.44	-	-	-	-	-	-	-	-	33.06	-
							109	L	26.21	33.45	19.62	28.70	29.20	-	-	-	-	-	25.49	33.75	19.25	28.39	29.12
							109	L	26.49	30.17	18.11	25.38	26.14	-	-	-	-	-	25.41	30.28	18.04	25.18	25.48
							109	D	No CT	35.88	28.35	33.28	33.09	-	-	-	-	-	36.82	-	28.72	32.92	33.18
							109	D	31.76	-	27.88	35.55	-	-	-	-	-	-	32.05	-	28.22	35.68	-
	6	02.08.2018	17.08.2018	21.12.2018	141	1026	102	L	-	-	-	-	31.56					34.18					-
							102	D	31.00	17.08	29.03	26.19	29.03	32.42	18.29	30.27	28.35	30.72	-	-	-	-	-
							102	D	33.24	36.05	32.79	35.33	-	-	-	-	-	-	-	-	-	-	-
				01.02.2019			102	D	29.94	27.82	23.06	27.12	27.50	-	-	-	-	-	30.95	28.07	23.10	26.94	27.69
				15.02.2019			102	D	-	25.93	36.04	30.61	34.62	-	-	-	-	-	-	-	-	32.40	-
							102	D	24.97	29.72	23.89	28.21	26.27	-	-	-	-	-	24.66	29.69	23.50	34.64	26.02

**Table 5:** Frequency of both genogroups at a fish-level (n = 67) according to seawater farms included in the monitoring period.

Farm		Both	EM-90-like	LF-89-like	Neither	Total	
						n	%
1		0	1	0	0	1	1.5
2		7	11	4	0	22	32.8
3		0	0	0	0	0	0.0
4		2	15	1	1	19	28.4
5		0	0	19	0	19	28.4
6		0	1	3	2	6	9.0
Total	n	9	28	27	3	67	100
	%	13.4	41.8	40.3	4.5	100	

**Table 6:** Frequency of both genogroups at the fish-level (n = 67) according to gross pathology severity.

Number of LF-89-like qPCR+ Tissues		Number of EM-90-like qPCR+ Tissues						Total	
		0	1	2	3	4	5	n	%
0		3	4	5	3	4	12	31	46.2
1		5	1	0	0	1	1	8	11.9
2		2	0	1	0	0	0	3	4.5
3		3	1	0	0	1	0	5	7.5
4		4	1	0	0	1	0	6	9.0
5		13	1	0	0	0	0	14	20.9
Total	n	30	8	6	3	7	13	67	100
	%	44.8	11.9	9.0	4.5	10.4	19.4	100	

**Table 7:** Frequency of both genogroups at a fish-level (n = 64) according to liver nodule severity. Three positive fish for universal *P. salmonis* qPCR but negative for genogroup-specific qPCR (Ct values ≥ 33.37 for EM-90-like and Ct value ≥ 34.54 for LF-89-like) were scored 0 for liver nodules and classified as “Neither”.

Liver Nodule Score		Both	EM-90-like	LF-89-like	Total	
					n	%
**0**		7	13	24	44	68.7
**1 (mild)**		1	3	2	6	9.4
**2 (moderate)**		1	8	1	1	15.6
**3 (severe)**		0	4	0	4	6.2
**Total**	**n**	9	28	27	64	100
	**%**	14.1	43.7	42.2	100	

## Data Availability

The data presented in this study are available on request from the corresponding author.

## Supplementary Materials

The following supporting information can be downloaded at: https://www.mdpi.com/article/10.3390/pathogens12030450/s1, Table S1: Semi-quantitative gross pathology classification system; Table S2: Semi-quantitative results obtained from the anatomopathological examination of each of the fish examined.
